# The Viscoelastic Behaviour of Waterlogged Archaeological Wood Treated with Methyltrimethoxysilane

**DOI:** 10.3390/ma14185150

**Published:** 2021-09-08

**Authors:** Magdalena Broda, Morwenna J. Spear, Simon F. Curling, Graham A. Ormondroyd

**Affiliations:** 1 Department of Wood Science and Thermal Techniques, Faculty of Forestry and Wood Technology, Poznań University of Life Sciences, Wojska Polskiego 38/42, 60-637 Poznań, Poland; 2 BioComposites Centre, Bangor University, Deiniol Road, Bangor, Gwynedd LL57 2UW, UK; m.j.spear@bangor.ac.uk (M.J.S.); s.curling@bangor.ac.uk (S.F.C.); g.ormondroyd@bangor.ac.uk (G.A.O.)

**Keywords:** archaeological wood, rheological behaviour, methyltrimethoxysilane, silane treatment, mechanical properties, DMA, wood conservation

## Abstract

Waterlogged wood treatment with methyltrimethoxysilane (MTMS) proved effective in stabilising wood dimensions upon drying (anti-shrink efficiency of 76–93%). Before the method can be proposed as a reliable conservation treatment, further research is required that includes the evaluation of the mechanical properties of treated wood. The aim of the study was to characterise the effect of the treatment on the viscoelastic behaviour of archaeological waterlogged elm and oak wood differing in the degree of degradation. Dynamic mechanical analysis in the temperature range from −150 to +150 °C was used for the study. To better understand the viscoelastic behaviour of the treated wood, pore structure and moisture properties were also investigated using Scanning Electron Microscopy, nitrogen sorption, and Dynamic Vapour Sorption. The results clearly show that methyltrimethoxysilane not only prevents collapse and distortions of the degraded cell walls and decreases wood hygroscopicity (by more than half for highly degraded wood), but also reinforces the mechanical strength by increasing stiffness and resistance to deformation for heavily degraded wood (with an increase in storage modulus). However, the MTMS also has a plasticising effect on treated wood, as observed in the increased value of loss modulus and introduction of a new tan δ peak). On the one hand, methyltrimethoxysilane reduces wood hygroscopicity that reflects in lower wood moisture content, thus limiting the plasticising effect of water on wood polymers, but on the other hand, as a polymer itself, it contributes to the viscous behaviour of the treated wood. Interestingly, the effect of silane differs with both the wood species and the degree of wood degradation.

## 1. Introduction

Archaeological waterlogged wooden artefacts are excavated mainly from wet ground or water reservoirs. Fully saturated with water from the surrounding environment of the burial site, they usually look good and retain their original shape and dimensions. However, due to microbial activity, the main wood chemical components (in the first instance, carbohydrates) degrade over time, making the cell walls thinner and weaker. Thus, when exposed to drying, waterlogged wood tends to irreversibly change its shape and dimensions due to shrinkage and cell wall collapse [1,2,3]. To preserve its integrity and prevent permanent destruction, the historical wooden objects therefore require immediate conservation treatment.

Currently, the most common solutions applied by conservators are based on polyethylene glycol (PEG). This is commercially available in a wide range of molecular weights, which makes it useful for the conservation of wood with varying degrees of degradation and permeability. PEG can penetrate waterlogged wood tissue and replace water molecules, thus reinforcing the wood structure and improving its dimensional stability [4,5,6,7]. However, it is not devoid of significant shortcomings, such as high leachability, and a tendency to be degraded to acidic by-products over time (which causes further chemical deterioration of the PEG impregnated wood) [8,9,10,11]. Additionally, the PEG-treated wood has increased hygroscopicity, resulting in strong swelling at high relative humidity, leading to irreversible wood degradation when strong swelling can induce cracking. It is also known that PEG has a plasticising effect on the treated wood [8,9,10,11]. Other chemicals that have been used so far for wood conservation, such as alum salts, carbohydrates, sugar alcohols, proteins, various resins or waxes, are not without drawbacks as well [12,13,14,15,16,17,18,19]. Therefore, the research continues on developing alternative wood consolidants, including those based on natural materials, such as cellulose or lignin derivatives, beeswax, colophony and other natural polymers, or using nanotechnology to create nanocomposites or supramolecular polymer networks [19,20,21,22,23,24,25,26,27,28,29,30,31,32,33].

Some recent studies aimed at the application of organosilicon compounds with various functional groups. Although the first attempts to use these chemicals in wood conservation already have a history [34,35,36], only recently has more comprehensive research been resumed to increase understanding of the properties of the treated wood [37]. One of the effective alkoxysilanes is methyltrimethoxysilane (MTMS). Although it can slightly lighten the original wood colour, it proved efficient in dimensional stabilisation of waterlogged wood, differing in the degree of degradation, ensuring its hydrophobisation and limitation of susceptibility to brown-rot decay [37,38,39]. However, before establishing this kind of treatment as a practical conservation method, the influence of the conservation agent on the physical and mechanical properties of the wood also have to be evaluated to ensure that it does not pose a threat to the integrity of the conserved object.

The mechanical performance of wood depends on many factors, including wood species, presence of juvenile or reaction wood, and density. The roles of temperature and moisture content at the time of testing [40,41,42,43,44] and the hierarchical structure of the wood are also recognised, both in terms of grain orientation during the test and at a micro-scale, considering the orientation of the microfibrils in the various cell wall layers [40,41,42,43,44,45,46,47]. Strength and stiffness are also dependent on any chemicals used for wood modification [48,49,50]. Methyltrimethoxysilane is also used as a compatibiliser within fibre-based composites, a coating for the nanocellulose scaffold or applied for wood treatment in combination with other chemicals, and has been proven to have a positive impact on the mechanical properties of the modified materials [51,52,53,54,55]. Therefore, a similar reinforcing effect on wood structure is expected when it is applied for treatment of waterlogged archaeological wood, which results in its successful dimensional stabilisation.

Dynamic Mechanical Analysis (DMA) has been used to study the viscoelastic behaviour of polymers and polymer composites. As wood is a composite material of three polymers (cellulose, hemicellulose and lignin), it has also been used in many studies on treated and untreated wood in both the wet state and the dry state [56,57,58,59,60,61]. Among other things, it has been employed to investigate the rheological behaviour of particular cell wall polymers [59,62,63], to measure the response of wood to changes in both temperature and humidity at the molecular or microstructural level [58,64,65], to study the decay processes in wood [66,67,68], to analyse mechanical properties of wood composites [69,70,71] or to assess the effect of different modifying agents on the mechanical properties of wood and wood-based composites [72,73,74]. DMA is also applicable for studying the viscoelastic behaviour of degraded archaeological wood (having altered the quantity and quality of the cell wall components), as well as for investigating the effect of different chemicals used for wood conservation. This knowledge is essential to predict the stability of wooden objects and properly design exhibit mounts and supports to ensure the safety of the artefact and fulfil the conservation requirements.

The aim of the research was to characterise the viscoelastic behaviour of archaeological waterlogged elm and oak wood differing in the degree of degradation and investigate the effect of methyltrimethoxysilane on its mechanical properties, which has never been studied before. The DMA thermal scan technique was applied to achieve this over the temperature range from −150 to +150 °C. Although the timber had been retrieved from anaerobic waterlogged conditions, it was carefully dried (by exchange with ethanol to minimise distortion effects), and DMA studies were carried out in the air dry state, i.e., at atmospheric relative humidity and room temperature. This reflects the objective of the MTMS modification, which is to enable artefacts from waterlogged conditions to be stored or displayed in museum collections under atmospheric conditions after a simple treatment. To better understand the viscoelastic behaviour of the treated wood, wood structure and moisture properties were also analysed using Scanning Electron Microscopy (SEM), surface area and pore volume measurements, as well as the Dynamic Vapour Sorption (DVS) method. MTMS efficiency in wood dimensional stabilisation along with its potentially positive influence on the mechanical behaviour of the treated wood could increase the prospects for future application of this chemical in conservation of wooden heritage.

## 2. Materials and Methods 

### 2.1. Materials

In the presented study, waterlogged archaeological oak (*Quercus robur* L.) and elm (*Ulmus* spp.) excavated from the sediments of the Lednica Lake in the Wielkopolska Region, Poland, were analysed. Dating back to the turn of the 10th and 11th centuries, the wooden logs looked well-preserved. However, the elm log and the outer part of oak (sapwood) were severely degraded (estimated loss of wood substance was about 70–80%). In contrast, the oak heartwood, characterised by a dense, hard texture, was decayed only to a limited extent, with loss of wood substance of about 25% [75,76].

Sound contemporary oak sapwood and heartwood, as well as elm heartwood, were sourced from commercial timber merchants and used for comparison.

### 2.2. Methods

#### 2.2.1. Sample Preparation

**Figure 1:**
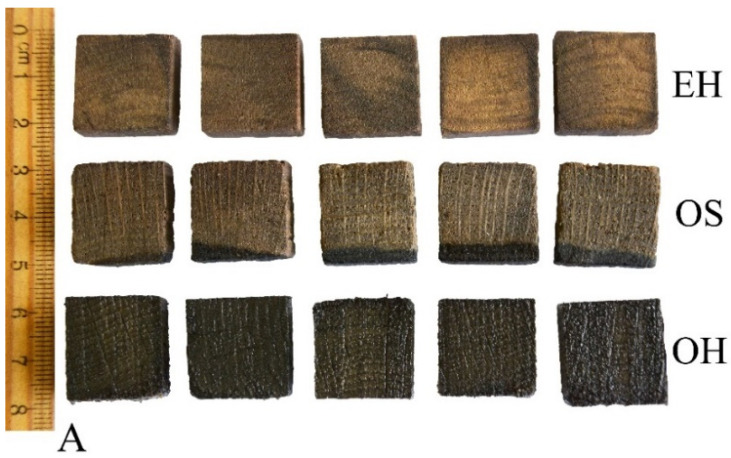

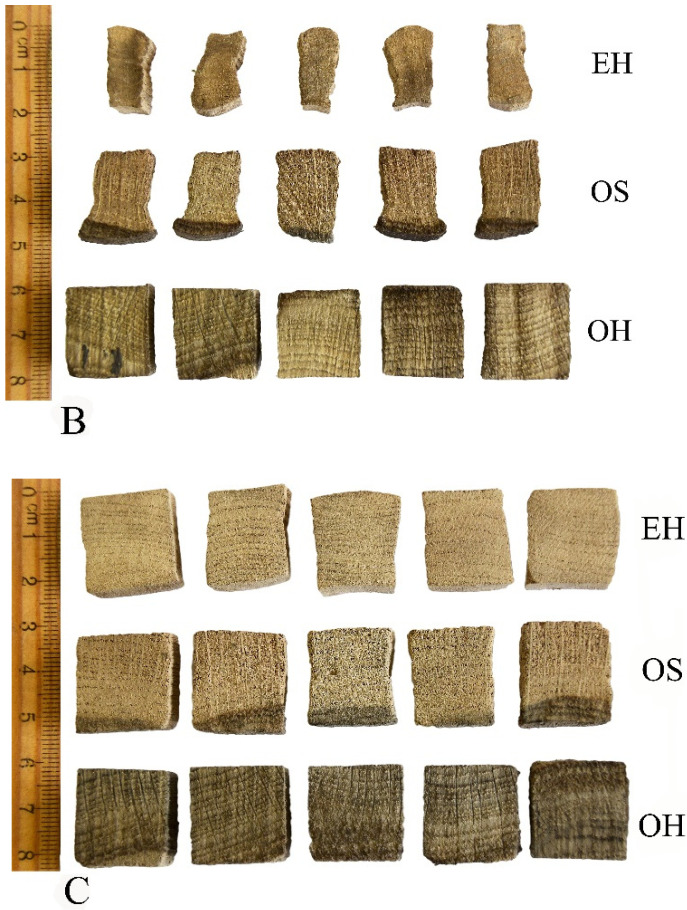
Archaeological wood samples: (**A**) waterlogged untreated and before any drying; (**B**) air-dried untreated; (**C**) air-dried MTMS-treated; EH—elm heartwood, OS—oak sapwood, OH—oak heartwood.

Small rectangular samples (20 mm × 20 mm × 10 mm in the radial, tangential and longitudinal directions, respectively) were cut out from particular zones of the waterlogged logs: sapwood and outer heartwood from oak and outer heartwood from elm (Figure 1A). To improve the effectiveness of a silane treatment, the samples were then dehydrated with 96% ethanol for four weeks [77]. Following dehydration, the specimens were divided into two sets consisting of 5 samples of each type. The first set was subjected to silane treatment with a solution of 50% methyltrimethoxysilane (MTMS) in 96% ethanol (*v*/*v*) using the oscillating pressure method (vacuum of 0.9 bar for 0.5 h and subsequent pressure of 10 bars for 6 h, repeated 6 times). A further 5 samples were used for moisture content determination without any treatment (see Section 2.2.2). After treatment, the specimens were removed from the silane solution and air-dried at ambient pressure and room temperature (25–28 °C) for 2 weeks (Figure 1B). The second set served as untreated controls, the samples removed from ethanol and air-dried as described above (Figure 1C). The dry untreated and treated wooden blocks thus obtained were then cut into smaller specimens with the dimensions appropriate for DMA measurements.

#### 2.2.2. Effectiveness of the Treatment Calculations

To evaluate the effectiveness of the MTMS treatment, the weight percent gain (WPG) was calculated according to Equation (1):

(1) $$\mathrm{WPG}=\frac{W_{t}-W_{0}}{W_{0}}\times 100$$

where W_0_ is the estimated oven-dry weight of the sample before impregnation and W_t_ is the oven-dry weight of the sample after treatment. Waterlogged wood specimens cannot be oven-dried before treatment (it would cause its shrinkage, cracking and irreversible deformation, and the sample would be useless for further study). Therefore, the oven-dry weight of the examined samples before treatment was calculated on the basis of the water content in similar samples that had been dried for moisture content determination (5 replicates were used for the measurement).

The moisture content (MC) at room temperature of treated and untreated wood was determined using the standard oven-drying method (105 °C) and calculated as a ratio between the mass of water to the mass of a dry sample. Additionally, the wood moisture content (MC) at the time of the DMA test was determined.

Based on the measurements of the pre- and post-treatment sample dimensions, wood volumetric shrinkage (S) and anti-shrink efficiency coefficient (ASE) were calculated according to Equations (2) and (3), respectively, in order to assess the effectiveness of the treatment for dimensional wood stabilisation:

(2) $$S=\frac{V_{0}-V_{1}}{V_{0}}\times 100$$

where V_0_ is the initial volume of the samples (in the waterlogged state) and V_1_ is the final volume of the dried samples. It was calculated for untreated and treated samples, to give S_u_ and S_t_, respectively, where S_u_ is the volumetric shrinkage of the untreated specimens and S_t_ is the volumetric shrinkage of the treated specimens, respectively.

(3) $$\mathrm{ASE}=\frac{S_{u}-S_{t}}{S_{u}}\times 100$$

The ASE value indicates what percentage of untreated wood shrinkage has been suppressed by the applied treatment. An ASE of 100% means that wood dimensions have not changed during drying. ASE values below 100% indicate wood shrinkage, with low values indicating very high magnitudes of shrinkage, while values above 100% point to swelling of the wood. A similar concept is used in wood modification technologies, but in a different context or a water soak/oven-dry test [78,79]. It should be remembered that this is a modification of archaeological wood to inhibit large magnitude shrinkage or deformation on drying rather than addressing a soaking-induced swelling under cyclic wetting.

Bulk density at the time of the test (ρ) was calculated as the ratio of the sample weight (prior to test) to its volume (prior to test). Thus, it is the bulk density after air-drying and conditioning under ambient temperature and relative humidity of about 20 °C and 50%.

#### 2.2.3. Dynamic Mechanical Analysis Measurements

Dynamic Mechanical Analysis (DMA) was performed on a Triton Technology DMA analyser (Grantham, UK) using a single cantilever deformation mode. The samples of dimensions ca. 20 mm × 10 mm × 3 mm (in radial, longitudinal and tangential directions, respectively) were mounted in a single cantilever clamp, resulting in the bending moment being applied in the tangential direction. The storage modulus (E′), loss modulus (E″) and tan δ (E″/E′) were measured to observe the relaxation behaviour of the sample over the temperature range from −150 to 150 °C with a heating rate of 5 °C/min, loading the dynamic force of 0.2–1 N oscillated with three different frequencies, 0.1, 1 and 10 Hz. The static force of 2 N was applied to the sample.

Five replicates of each sample type (elm, oak sapwood, oak heartwood, contemporary untreated, archaeological untreated and treated with MTMS) were measured. In each group, the samples had all been cut from the same wooden block. The storage modulus (E′), the loss modulus (E″) and the loss factor (tan δ = E″/E′) were determined by the DMA. 

The data obtained were statistically analysed using Statistica 13.3 software (TIBCO Software Inc., Palo Alto, CA, USA); post hoc Tukey’s honestly significant difference (HSD) test was applied to find mean values of individual mechanical parameters that differ significantly within specific wood type groups (elm heartwood, oak sapwood, oak heartwood) and hence to evaluate the effect of silane treatment on wood viscoelastic behaviour.

#### 2.2.4. Scanning Electron Microscopy Imaging

To facilitate interpretation of DMA results, the wood microstructure was analysed using a Quanta FEG 650 Scanning Electron Microscope (FEI Company, Hillsboro, OR, USA) and a Scanning Electron Microscope JEOL 7001F with a Secondary Electron Imaging (SEI) detector (JEOL, Tokyo, Japan). Dry wood samples were cut into smaller pieces. Their cross-sections were coated with a 15 nm layer of carbon (oak samples) or chromium (elm wood) using a high vacuum coating system Leica EM ACE600 (Leica Microsystems GmbH, Wetzlar, Germany). Then, the samples were mounted in the specimen holder and analysed at 1, 5 or 15 kV, depending on the sample.

#### 2.2.5. Surface Area and Pore Volume Measurements

Changes in the wood cell wall porosity after the MTMS treatment were characterised using a Gemini Surface Area Analyser (Micromeritics Instrument Corporation, GA, USA) and a nitrogen absorption method. Wood samples were de-gassed and placed in the glass tubes; then, the nitrogen sorption isotherms were recorded at liquid nitrogen temperature. Based on the Brunauer–Emmet–Teller (BET) theory [80], i.e., the volume of nitrogen absorbed on the surface of the cell walls at different partial pressures, the surface area was calculated using Micromeritics Stardriver software. Wood porosity was analysed using the Barrett–Joyner–Halenda (BJH) method [81], which is appropriate for making comparisons between analysed samples and gives reliable results for meso- and macropores with diameters above 4 nm [39,82].

#### 2.2.6. Moisture Sorption Analyses

To better understand the viscoelastic behaviour of the studied material, wood moisture properties were analysed using a Dynamic Vapour Sorption (DVS) system (Surface Measurement Systems, London, UK). About 10 mg of powdered wood of each type was analysed. The measurements were performed at a constant temperature of 21 ± 0.2 °C. The flow rate of nitrogen passing over the sample was adjusted to 200 cm^3^ min^−1^. The device was scheduled to start at 0% of air relative humidity (RH) and then increase in 10% steps up to 95% RH for the adsorption, and the reverse was carried out for the desorption phase. All the data, i.e., running time, sample mass change, target and actual RH, were recorded every 20 s. Then, they were used in the analysis of the isotherms obtained. During the measurement, the equipment maintained the sample at a constant RH until the equilibrium was reached (i.e., the ratio of change in mass to change in time remained less than 0.002% per minute for at least 10 min). However, Glass et al. [83] reported that much longer hold times of the equilibrium change over point can produce slightly different final moisture content values. However, for comparison of the moisture properties of wood samples in this study, the stated equilibrium point was chosen as a compromise between the rational analysis test length and the sufficient accuracy of the moisture content.

## 3. Results and Discussion

### 3.1. Effectiveness of the Treatment and Its Impact on the Wood Structure

Treatment of waterlogged archaeological wood with methyltrimethoxysilane resulted in improved dimensional stabilisation after air-drying, which is consistent with the previously reported results [75,84]. As is clear from Table 1, the highest shrinkage was observed for untreated wood, especially the highly degraded elm heartwood (about 70%) and oak sapwood (about 45%), while with treated specimens, the shrinkage was significantly reduced (about 6–16% for the most degraded samples and less than 2% for a better-preserved oak heartwood). As a consequence, high ASE values were calculated for all MTMS-treated woods. The highest anti-shrink efficiency was obtained for oak heartwood, while oak and elm sapwood coefficients were lower by about 7 and 17%, respectively.

**Figure 2:**
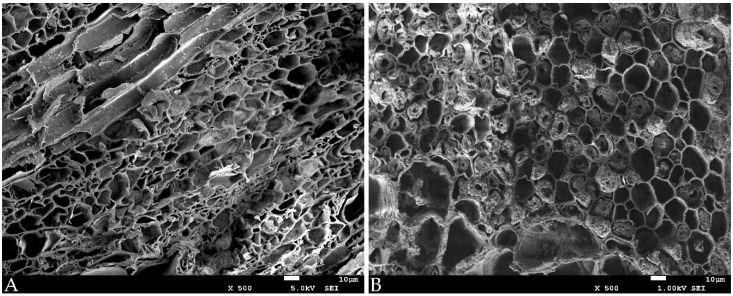
SEM images of air-dried archaeological elm samples: (**A**): untreated; (**B**): treated with MTMS.

**Figure 3:**
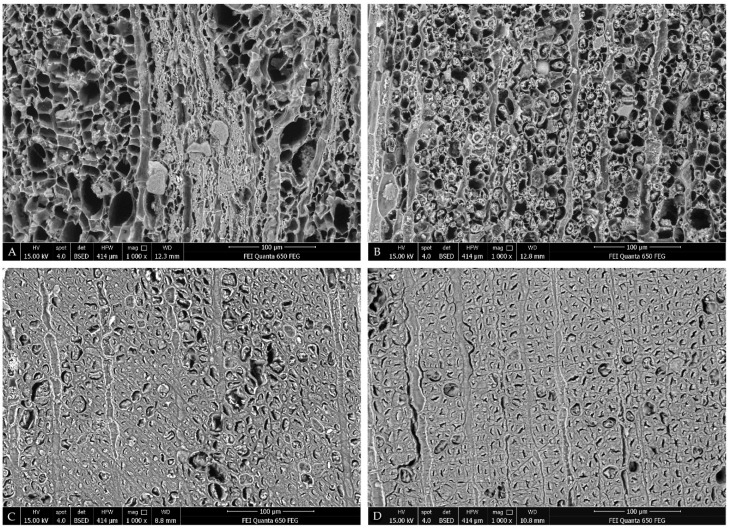
SEM images of air-dried archaeological oak samples: (**A**): untreated sapwood; (**B**): sapwood treated with MTMS; (**C**): untreated heartwood; (**D**): heartwood treated with MTMS.

The effect of the MTMS treatment is well visible on the macro-scale in Figure 1. The change in wood dimensions between the wet (1A) and the air-dried state (1B) of untreated samples is particularly pronounced for the most degraded elm heartwood (EH). For oak sapwood (OS), however, despite the similar degree of wood degradation (about 70–80%), the shrinkage is smaller due to the differences in anatomical structure between the two species (e.g., the presence and dimensions of rays, cell types and the cell wall thicknesses). The applied treatment resulted in the significant improvement of dimensional wood stability upon drying—the dimensions of dry treated samples (Figure 1C) are more similar to the dimensions of wet samples. The effect described is also visible on the micro-scale in SEM images (Figure 2 and Figure 3), especially for the most degraded elm heartwood and oak sapwood. In untreated air-dried samples (Figure 2A and Figure 3A), the cells are of irregular shape, often flattened, with the thin, rolling cell walls, while for the treated ones, with reduced shrinkage (Figure 2B and Figure 3B), the cells are more regular and not flattened, similar to sound wood. The differences in the microstructure between untreated and treated archaeological oak heartwood are hardly recognisable in SEM pictures (Figure 3C,D).

Analysis of cell wall pores clearly showed differences between contemporary and archaeological samples (Table 2) resulting from wood degradation, i.e., a higher porosity of archaeological wood. It also revealed further differences in structure between untreated and treated degraded wood. Generally, despite the different shrinkage levels, silane treatment decreased surface area and total pore volume of the cell walls proportionally to the amount of a chemical deposited in wood. The only exception was the most degraded elm samples with the highest shrinkage level (almost 70%), where the collapse of the cell walls reduced their porosity so significantly that the effect of treatment is unnoticeable. The change in porosity is followed by the change in bulk density (except AET, as described above).

The decrease in bulk density of archaeological elm on treatment with MTMS can be explained with reference to the WPG data, shrinkage coefficient and the SEM micrographs. To demonstrate a decrease in density, the volume increase by the sample must exceed the gain in weight from the MTMS treatment. This is the case for AET, as the WPG was smaller than AOST (173% vs. 203%), yet avoided shrinkage was high (52.4% by subtraction, Table 1, compared with 37.4% for AOST). For further consideration, we know the MTMS must be deposited on available surfaces within the two kinds of wood. Looking to Figure 2B, we observe that in AET the compound middle lamella remains intact, and a small quantity of degraded secondary cell wall material as a porous aggregate can be found in the cell lumen spaces. These aggregates are fewer in number and smaller in quantity than the equivalent material in the AOST material shown in Figure 3B. Therefore, while both AET and AOST have significant quantities of porous aggregates in cell lumina, the AOST presents a greater surface area of this material for the MTMS additional WPG to be accommodated with limited extra benefit on swelling or stiffness. By comparison, the MTMS deposition into the compound middle lamella results in the retention of cell shape, as commented previously, and the quantities of middle lamella remaining is more consistent between the two species. Thus, the AOST material undergoes an increase in density while AET shows a decrease.

### 3.2. The Effect of the Treatment on the Moisture Properties of Archaeological Wood

**Figure 4:**
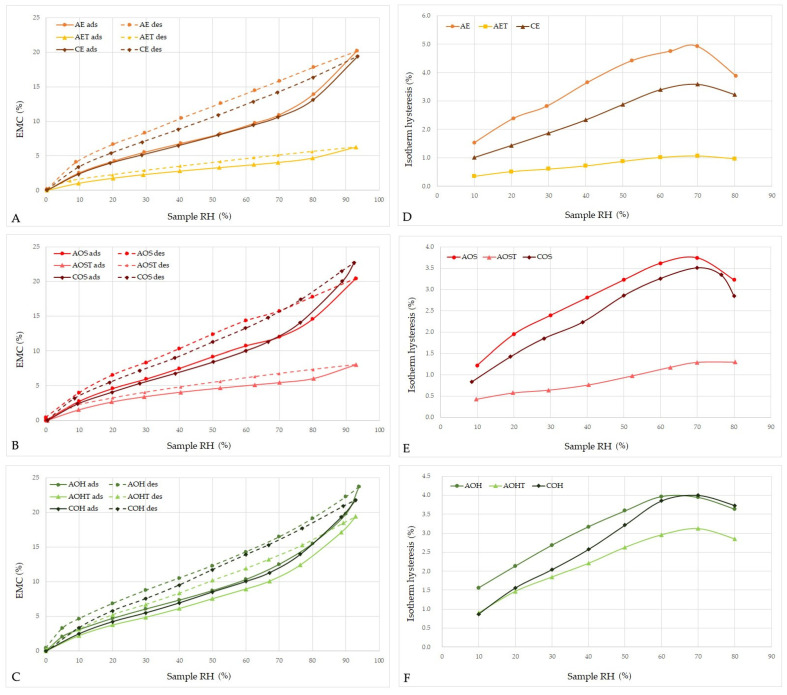
Sorption isotherms (**A**–**C**) and hysteresis (**D**–**F**) for air-dried contemporary and archaeological elm (**A**,**D**) and oak (**B**,**C**,**E**,**F**) wood samples (untreated and treated with MTMS); ads—adsorption, des—desorption.

Treatment with MTMS significantly changed the moisture properties of archaeological wood. The results of DVS analysis, presented as sorption isotherms and hysteresis in Figure 4, show a decrease in maximum equilibrium moisture content (EMC_max_) and hysteresis for all the treated samples, which is in line with the results reported previously [39]. The most pronounced decrease in EMC_max_ after treatment can be seen for elm heartwood and oak sapwood (from 20.2 to 6.3% for elm, and from 20.4 to 8% for oak sapwood). This results from the high quantity of silane deposited in wood during the treatment (WPG was about 200%, see Table 1). However, the change in sorption properties is also visible for oak heartwood (EMC_max_ changed from 23.7 to 19.4%), but it is proportionally smaller due to the lower silane content. It has already been shown that methyltrimethoxysilane can chemically react with hydroxyls present on wood polymers, hence the observed hydrophobising effect on wood, which is higher the more that silane molecules are able to interact with wood hydroxyls [85].

### 3.3. Viscoelastic Behaviour of Untreated and MTMS-Treated Archaeological Wood

It is well-known that the treatment of lignocellulosic materials with different chemicals often leads to their softening [27,86,87]. Therefore, MTMS applied for the conservation of waterlogged archaeological wood could have a similar effect, and this study sought to quantify this. The results obtained show that the silane limits wood hygroscopicity, leading to the reduced wood moisture content (Table 1). It is possible that this decreases the well-known plasticising effect of water in wood cell walls, which may compensate for any newly induced plasticising effect of the polymer itself. As a result, it is important to consider these conflicting effects of the wood modification process.

The dynamic mechanical analysis allows the quantification of several phenomena, contributing to new understanding in this area. The most commonly discussed is the glass transition temperature (T_g_), i.e., the transition from a glassy state at lower temperatures to a rubbery state above T_g_. In wood, there may be T_g_ events relating to the lignin, and the polysaccharides (amorphous cellulose and hemicelluloses) [59]. The T_g_ of all the wood polymers has been shown to be strongly changed by the presence of moisture, when studied in isolation [88,89]. Secondly, the secondary relaxations can be observed for the polymers in the glassy state (i.e., below T_g_), which relate to motions of small chain segments or rotation of functional groups within the polymer chain [60,90]. Lignin, hemicellulose and cellulose all demonstrate secondary relaxations when studied ex situ [91,92,93]. 

**Figure 5:**
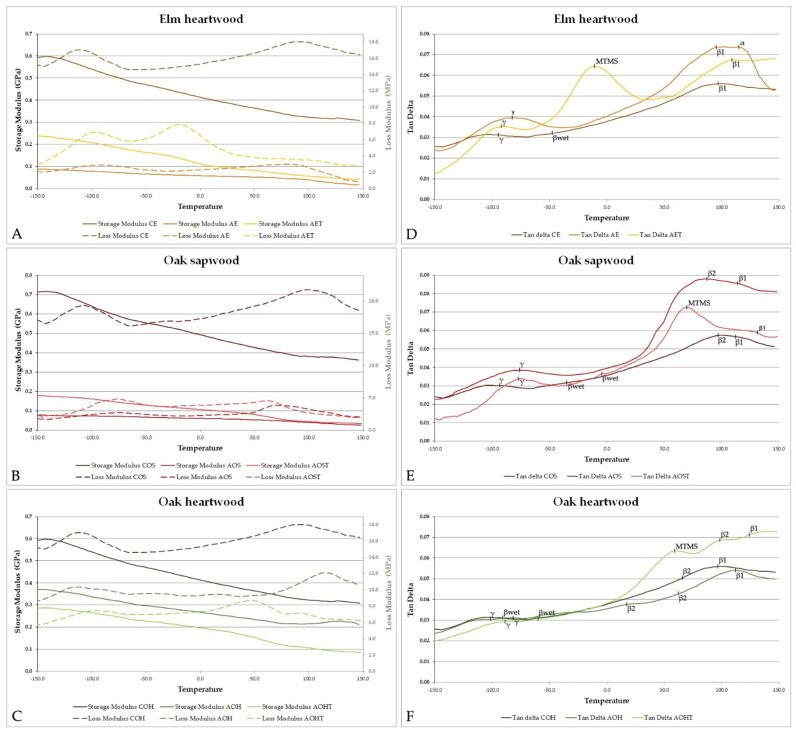
Comparison of storage modulus (E′), loss modulus (E″) (**A**–**C**) and loss factor (tan delta) (**D**–**F**) exemplary graphs for air-dried contemporary, archaeological untreated and MTMS-treated elm and oak wood with mean values of specific tan delta peaks marked.

Samples of the contemporary and archaeological elm and oak with similar dimensions were analysed using DMA in the temperature range −150–150 °C. Example DMA scans for all studied wood samples are presented in Figure 5. Although E′, E″ and tan δ graphs for all the contemporary wood specimens are relatively similar, they differ significantly from their archaeological untreated and treated counterparts and show small differences between wood species. Although all the samples were stored in identical conditions before the measurements, their moisture contents were different depending on wood type, the degree of degradation and the treatment applied (Table 3). MTMS treatment significantly reduced the MC of archaeological elm and oak sapwood samples to about 4%. Higher MC was observed for untreated oak heartwood specimens (6.8 and 7.7%), but for the treated samples, due to the better state of their preservation (resulting in lower permeability and thus lower WPG of silane), only a moderate MC reduction was noted (to about 5.2%).

Tan δ graphs enable individual secondary relaxations peaks for wood polymers to be recognised in the samples studied (Figure 5B,D,F). Differences between the rheological behaviour of contemporary and archaeological wood in the example of elm have already been described by Spear and Broda [77]. In general, for contemporary samples, the peak at about −100 °C refers to the γ-relaxation and is associated with rotations of methylol groups present on wood polysaccharides (hemicelluloses and amorphous regions in cellulose) [60,90,94]. In degraded samples, where polysaccharide content is reduced, resulting in a lower ability to bind water molecules, this peak is shifted towards higher values [60,77], which is visible for elm heartwood and oak sapwood. For the well-preserved archaeological oak heartwood, the position of this relaxation remained similar to contemporary oak wood.

The tan δ peaks in the higher temperature range are commonly identified as the β-peak, which is a secondary relaxation relating to segments of the polymer chain, and the α-peak or glass transition temperature. The β-peak typically occurs between −7 and +34 °C in wood containing low to moderate quantities of moisture [95], but has also been reported over a much wider temperature range (e.g., −53 to +53 °C [96]) and as high as 70 °C [59] or 83 °C [97], and up to 118 °C in oven-dried wood [98]. A separate β_wet_ peak has been observed by some researchers [99]. The α-peak relates to micro-Brownian motions of the polymer chain as the material moves from glassy to viscous state, and typically occurs at high temperatures (150 to 250 °C) for air dry and oven-dry wood [96,98,100]. 

In this study, some differences in the location of these peaks were seen between the three undegraded contemporary woods. In all three undegraded woods, the β_wet_-peak was very small, and occurred near −0.7 to +21 °C in CE, −9 to +41 °C for COS and −9 to +18 °C in COH; this variation related to differences between samples and the frequency used. There was a stronger peak at 93–114 °C for CE, 96–114 °C for COS and 91–111 °C for COH, which in some cases (but not all) was associated with a decrease in E′, which could be attributed to α-peak or glass transition. Where there was no substantial decrease in the E′ value, and the tan δ graph indicated an upturn at temperatures approaching 150 °C (the end of temperature scan range), it could be argued that this peak is a β-peak not an α-peak [97]. In the archaeological samples, the tan δ peaks were stronger than the contemporary wood samples, most notably the β-peaks at the higher temperature range. In several cases, it was noted that peaks in this region were broad, potentially incorporating two poorly defined superimposed peaks. The main difference between the contemporary and archaeological wood is the removal of polysaccharides, so it is expected that the DMA output is dominated by molecular relaxations of lignin, with only a minor contribution from degraded hemicellulose or cellulose. The additional peak in the AE sample at 114 °C with an associated strong decrease in E′ could therefore be a glass transition, brought to lower temperature as a result of degradation. It should be noted that due to the influence of transient moisture effects, peak information in this range is difficult to definitively interpret. Therefore, the data in this higher temperature range are presented here only to permit comparison with the degraded and MTMS treated samples, where changes relative to this baseline are the object of study.

Figure 5D shows that a strong additional peak was present for the MTMS-treated archaeological elm wood (AET). This peak occurred at −5 °C, which is close to the location where the β_wet_-peak often occurs. The peak in tan δ was accompanied by a substantial decrease in E′, possibly indicating that the presence of MTMS plasticised the degraded wood or underwent a glass transition event itself. However, it was surprising to note that the newly introduced peaks in treated archaeological oak samples (AOST and AOHT, Figure 5E,F) did not occur at this temperature, but at higher values (70 and 58 °C, respectively). In both of the treated oak samples, the tan delta event was accompanied by a relatively strong decrease in E′, in the same manner as seen for the AET samples. In the treated oak samples, this relaxation event or glass transition lay closer to the temperature where the β-peak usually occurs. The difference in MTMS-peak temperature cannot relate to the level of wood degradation, as the AOS material was degraded to a similar extent to the AE, whereas AOH was less degraded. It seems likely that species difference (i.e., wood anatomy and proportions of cell types, or cell wall chemistry) contributes to this difference between MTMS treatment effect on the two timber species. The difference between MTMS peak temperature in archaeological elm and oak could have similar origins to the difference in porosity and surface area between these species (Table 2).

The origin of the MTMS peak and the associated loss of storage modulus at this temperature for treated archaeological wood are interesting, and could be a plasticisation effect. The presence of moisture in wood is well-known to act as a plasticiser, and increasing moisture content has the effect of reducing the temperature that T_g_ is observed for each of the wood components, as demonstrated by Goring, as early as 1963 [101,102]. This result has also been shown in DMA studies, reducing T_g_ from 130–205 °C for dry lignin to 80 °C for saturated lignin [89,96]. Studies on hemicelluloses reveal a shift of T_g_ from 150–220 °C in the dry state to 80 °C at 15% moisture content, and circa 20 °C at higher moisture contents (20–30%) [89,95,103]. Water-saturated samples are thus sometimes used to lower T_g_ to permit DMA measurement without incurring thermal degradation [56,57]. Other plasticisers such as polyethylene glycol and N-methyl-2-pyrrolidone (NMP) have been used to conduct immersion DMA experiments, offering a wider temperature range than for water, while again lowering the temperature at which T_g_ is observed [104,105]. However, it would be expected that the MTMS, if acting as a plasticiser and present in abundance within the treated samples, would lead to a peak at a similar temperature for the three treated woods. In a non-immersion experiment, using PEG-treated wood in atmospheric conditions, a reduction in the temperature of the γ-peak (i.e., plasticisation) and a new peak at 27 °C was reported by Obataya et al. [60]. The new peak was proposed to relate to motions within the backbone of the PEG polymer. This supports the hypothesis that the new MTMS peaks observed in this study relate to the grafted methyl trimethoxy units. 

For comparison, many other wood modification systems are not used specifically for a plasticising effect; however, they do have an effect on DMA observations, such as introducing new peaks relating to the polymerised new reagent (e.g., PMMA impregnation), or altering the location of T_g_ (e.g., PEG impregnation treatments [60], acetylation reactions [106]), or altering intensity and location of secondary relaxations (γ and β), e.g., acetylation and formaldehyde modifications [60,106]. For example, in acetylated wood, the γ-peak moves to a higher temperature and becomes broader but with a lower value for tan δ, and activation energy for this motion increases compared to untreated wood. This was proposed to relate to the substitution of acetyl groups for the hydroxyl site within the wood, leading to a reduction in methylol groups and an increase in acetyl groups, which have a similar relaxation motion. By contrast, formaldehyde treatment, which promotes cross-linking within the polysaccharide components, showed little change in γ-peak location, and no significant change in activation energy compared to untreated wood. The cross-linking reaction does not alter the methylol groups, which are responsible for the γ-relaxation.

DMA thermal scans of the studied wood samples enabled the determination of their mechanical parameters (storage modulus and loss modulus) at 25 °C (Table 3). First of all, except for oak heartwood, the storage modulus of archaeological wood was significantly lower in comparison with contemporary wood, which reflects its calculated loss of wood substance (about 70–80%), the resulting lower density and reduced cellulose and hemicelluloses content. It is well-known that wood mechanical parameters strongly depend on density, as well as on cellulose and hemicellulose contents in the cell wall, which account for wood stiffness and mechanical strength [67,77,107]. The density of COH examined in the research turned out to be very low for this wood species; moreover, the degree of degradation of AOH was only about 25%; hence, no significant differences in mechanical parameters were observed between them.

The effect of MTMS treatment is clearly visible for elm heartwood and oak sapwood, as E′ and E″ significantly increased compared to the values for untreated archaeological material. The highly degraded archaeological wood tissue soaked up large amounts of silane (WPG of about 200%). By covering and encrusting wood cell walls, MTMS reinforced their mechanical strengths by increasing their stiffness and resistance to deformation, which is seen in the increase in the E′ value. However, its plasticising effect can also be observed in an increased value of the loss modulus. On the one hand, MTMS reduced wood hygroscopicity that reflects in lower wood moisture content, thus limiting the plasticising effect of water on wood polymers, but on the other hand, as a polymer itself, it contributed to the viscous behaviour of the treated wood. Mechanical parameters of archaeological oak heartwood, however, remained almost unchanged after the treatment (the observed differences are not statistically important). It can be explained by a much lower amount of silane absorbed by the wood due its good state of preservation and thus lower permeability in comparison with highly degraded archaeological oak sapwood and elm heartwood.

To perform Arrhenius analysis, one sample of each wood type was additionally run on the DMA using three different frequencies (1, 5 and 10 Hz) but under the same loading conditions. The results are presented in Table 4. The increase in temperature seen for most tan δ peaks as the frequency is increased can be used to calculate the activation energy for the molecular motion associated with this tan δ event. The activation energy for the γ-peak (methylol groups) in contemporary wood was relatively consistent, calculated as 39.2, 31.0 and 38.4 kJ·mol^−1^ for elm, oak sapwood and oak heartwood, respectively (Table 4). This is in good agreement with values reported previously [72] and by other researchers [60] for the magnitude of E_a_ of γ peaks in wood. 

For the archaeological wood samples, the values of E_a_ of the γ-peak were more variable. Values of 62.4 kJ·mol^−1^ for AE, 98.7 kJ·mol^−1^ for AOS and 28.1 kJ·mol^−1^ for AOH indicate an increase for the heavily degraded samples, whereas the less-degraded oak heartwood showed a decrease compared to contemporary wood. The ranking of these activation energies reflected the degree by which the tan δ peak had been shifted to a higher temperature by the degradation of polymers, i.e., AOH with the lowest degradation and negligible shift of temperature had the lowest E_a_ value, while AOS with the highest shift in temperature had the greatest E_a_ value.

Treatment of the archaeological wood with MTMS led to a more consistent E_a_ value for the γ-peak, with 55.1, 46.1 and 45.5 kJ·mol^−1^ reported for AE, AOS and AOH, respectively. It appears that while MTMS alters the activation energy for rotation of methylol groups within archaeological wood, it does not give the same value as unmodified contemporary wood. This may be a result of the influence of the silane on the ability of ungrafted methylol units to rotate, for example, steric hindrance or altered electromagnetic charge.

Other researchers have shown that the activation energy of secondary relaxations is typically reduced by the presence of a plasticiser. For example, in PEG treated wood, Obataya et al. [60] reported a value of 36 kJ·mol^−1^ for the γ-relaxation (rotation of methylol groups) compared to 42 kJ·mol^−1^ for untreated wood. In this present study, the heavily degraded archaeological woods had an increased temperature for γ-peak (−75 to −50 °C, AE and AOS) than the contemporary woods (−100 to −79 °C), and the MTMS treated archaeological woods showed a reduction in temperature (−86 to −65 °C). The increase in T_γ_ was accompanied by an increase in E_a_ in the archaeological woods (62.4 and 98.7 kJ·mol^−1^ for AE and AOS, respectively), while the decrease in T_γ_ was accompanied by a decrease in E_a_ for treated archaeological woods (55.1 and 46.1 kJ·mol^−1^ for AET and AOST, respectively), returning to a value closer to that observed for contemporary wood (39.2 and 31.0 kJ·mol^−1^ for CE and COS). This is consistent with degradation-induced loss of mobility in the archaeological woods, and restoration of some of this mobility in the presence of the MTMS treatment.

The behaviour of the MTMS peaks was also of interest, but Arrhenius analysis may be negatively influenced by the superimposition of these peaks upon existing peaks of the wood cell wall polymers. The MTMS peak in AET gave the clearest result with an E_a_ value of 152.2 kJ·mol^−1^ for the peak at −5 to +5 °C. This activation energy value is in a similar range to that reported for β secondary relaxations, but not as high as would be expected for a glass transition temperature [100]. The effect of plasticisers on β secondary relaxations is complex [108], and further investigation is needed. By comparison, the E_a_ value for AOST was very high, 980.5 kJ·mol^−1^, and related to a peak at approx. 70 °C. This is close to the location of the beta peak in AOS samples. The definition of the peak in AOST, and the shift to a lower temperature value compared to the beta peak in the untreated sample were taken as a clear indication that this is the influence of MTMS treatment. Whether this peak relates to plasticisation or is due to the motion of the grafted MTMS monomer or its constituent parts was unconfirmed. The high value of E_a_ for this sample was consistent with the strong decrease in the E′ value at this temperature, taken as further confirmation that MTMS has in some way plasticised the archaeological oak wood, as similar values were reported for the α-peak in studies on dried wood by Jiang et al. [100]. The typical action of a plasticiser or diluent is to reduce the temperature of T_g_, as the small diluent molecules infiltrate the polymer and increase the available free volume for segmental motions [108]. 

The E_a_ value for the MTMS peak in the AOHT sample was 221.8 kJ·mol^−1^, and the peak occurred at approx. 73 °C. The lower value for this E_a_ may relate to measurement error in the peak of each tan δ curve, either resulting from measurement “noisE″ or the effect of superimposition on an area of the curve which contained several pre-existing peaks. However, the E′ values again decreased significantly at this point in the thermal scan, indicating plasticisation by the MTMS. It is not clear whether the T_g_ in AOST and AOHT related to the degraded polysaccharide component of the wood wall, or the lignin, however the occurrence of further tan δ peaks at higher temperatures (Table 3) indicates that only one component of the wood cell wall had been plasticised, and the other(s) remained in the glassy state at higher temperatures.

One aspect of interest is that the MTMS peaks did not occur at the same temperature in the two species. The lack of one specific temperature signature indicates that the new peaks are not related to one single functional unit within the MTMS structure, but instead may indicate that the MTMS plasticises existing motions of the wood polymers and is thus governed by the spatial location of these polymers in the two species. For elm, this gave a lower temperature MTMS peak than for oak. It could be proposed therefore that the structure of degraded elm in some way favours molecular motions of a specific kind, while in oak the MTMS plasticises a different motion or different chain segment, possibly the plasticisation of the T_g_ event itself, as indicated by the activation energy. 

## 4. Conclusions

MTMS treatment significantly reduced the shrinkage of archaeological wood on drying from the waterlogged state. ASE values of 92.8, 86.2 and 76.2% were observed for AET, AOST and AOHT, respectively. The intended location of the MTMS is the reactive sites on the wood cell wall material and the location of MTMS here prevents cell wall distortion and collapse during drying. However, in the heavily degraded samples, high weight gains were seen (172.8% for AE and 203.2% for AOS), and many MTMS monomers apparently also reacted onto the amorphous residues located inside cell lumina (remnants of the secondary cell wall, commonly seen in bacterial-degraded wood with high porosity). Thus, the WPG values for AET and AOST were very high, compared to 50% WPG observed in AOHT, where more secondary cell walls remained intact and attached to the cell wall with lower degradation and lower porosity values.

Cell wall collapse and distortion were avoided by the MTMS treatment, and in the two most heavily degraded woods (AET and AOST), this gave an increase in storage modulus at 25 °C in DMA experiments. DMA revealed a new peak in tan δ for the MTMS treated samples. This occurred at −5 °C for AET and between 58 and 70 °C for AOST and AOHT. The difference in location of the MTMS peak was attributed to differences in the architecture of the remaining cell wall materials between the two species. In the case of AOST and AOHT, there was a clear indication from the change in storage modulus at the same temperature as the MTMS peak, showing that a glass transition had even been plasticised by the presence of the grafted silanes. It is unclear whether this T_g_ is related to the degraded polysaccharide component of the wood wall or the lignin, and further research is required.

The effect of MTMS treatment turned out to be quite a complex problem considering its direct plasticising effect on wood and the simultaneously provided reduction in wood equilibrium moisture content, which reduces the plasticising effect of water on wood polymers. Moreover, the effect differed with both the wood species and the degree of wood degradation. Further study is then necessary, including nanoindentation measurements under different moisture conditions, to better understand the observed phenomena and establish the storage and exhibition conditions that would be safe for the mechanical stability of the silane-treated waterlogged wooden artefacts. Furthermore, it is essential to know if the stabilising effect of silane treatment goes along with the reinforcement of the cell wall of degraded wood. The increased knowledge about the effect of polymers such as MTMS on the wood properties will allow designing more efficient conservation agents targeted to the needs of specific wooden objects. 

## Figures and Tables

**Table 1:** Average values of the moisture content (MC) at room temperature, weight percent gain (WPG), volumetric shrinkage (S) and volumetric anti-shrink efficiency (ASE) of archaeological air-dried elm heartwood (AE), oak sapwood (AOS) and oak heartwood (AOH) untreated and treated with MTMS.

Wood Type	Treatment	MC (%)	WPG (%)	S (%)	ASE (%)
AE	untreated	9.2	-	68.7 ± 1.2	-
	MTMS	6.5	172.8 ± 4.16	16.3 ± 3.8	76.2 ± 5.5
AOS	untreated	9.0	-	43.4 ± 1.8	-
	MTMS	5.5	203.2 ± 15.04	6.0 ± 4.1	86.2 ± 9.4
AOH	untreated	9.7	-	23.2 ± 2.7	-
	MTMS	7.4	50.07 ± 1.64	1.7 ± 0.5	92.8 ± 1.5

**Table 2:** Average values of the surface area, total pore volume and bulk density of contemporary and archaeological wood samples untreated and treated with MTMS; ±standard deviation, archaeological untreated elm heartwood (AE), oak sapwood (AOS) and oak heartwood (AOH), archaeological MTMS-treated elm heartwood (AET), oak sapwood (AOST) and oak heartwood (AOHT), contemporary elm heartwood (CE), oak sapwood (COS) and oak heartwood (COH).

Wood Species	Wood ID	Surface Area (m^2^ g^–1^)	Total Pore Volume (cm^3^ g^–1^)	Bulk Density (g cm^–3^)
Elm heartwood	AE	1.60 ± 0.06	0.0038	0.53 ± 0.09
	AET	1.68 ± 0.03	0.0048	0.48 ± 0.01
	CE	0.53 ± 0.01	0.0016	0.71 ± 0.01
Oak sapwood	AOS	4.14 ± 0.10	0.0146 [39]	0.27 ± 0.03
	AOST	1.09 ± 0.07	0.0020 [39]	0.47 ± 0.01
	COS	0.60 ± 0.06	0.0012	0.67 ± 0.00
Oak heartwood	AOH	0.64 ± 0.01	0.0019	0.70 ± 0.01
	AOHT	0.30 ± 0.02	0.0014 [39]	0.73 ± 0.03
	COH	0.35 ± 0.02	0.0011 [39]	0.60 ± 0.01

**Table 3:** Average values of MC for DMA samples at the start of the measurement, bulk density of wood samples (ρ), temperature of tan δ responses, and E′, E″ and tan δ measured at 25 °C and a frequency of 1 Hz.

Wood ID	MC (%)	ρ (g cm^−3^)	Tan δ Response (°C)						E′ at 25 °C (MPa)	E″ at 25 °C (MPa)	Tan δ at 25 °C
			γ	MTMS	β_wet_	β_2_	β_1_	α			
CE	8.3 ± 0.3	0.71 ± 0.01 ^b^	−97 ± 2	-	−41 ± 15	-	97 ± 3**b**	-	394.0 ± 64.9 ^b^	15.3 ± 1.5 ^b^	0.039 ± 0.003 ^a^
AE	8.0 ± 0.4	0.53 ± 0.09 ^a(a)^	−86 ± 3	-	-	-	95 ± 2**b**	114 ± 2**b**	61.6 ± 5.5 ^a(a)^	2.8 ± 0.5 ^a(a)^	0.045 ± 0.005 ^ab(a)^
AET	4.2 ± 0.2	0.48 ± 0.01 ^a(a)^	−89 ± 3	−5 ± 8	-	-	109 ± 8	incr.	87.9 ± 4.1 ^a(b)^	4.8 ± 0.5 ^a(b)^	0.055 ± 0.006 ^b(a)^
COS	7.8 ± 0.2	0.67 ± 0.00 ^c^	−99 ± 0	-	−43 ± 5	98 ± 2**b**	110 ± 5**b**	incr.	439.2 ± 18.4 ^c^	15.8 ± 1.5 ^b^	0.036 ± 0.002 ^a^
AOS	8.4 ± 0.4	0.27 ± 0.03 ^a(a)^	−75 ± 4	-	-	93 ± 6	117 ± 0**b**	-	52.8 ± 8.2 ^a(a)^	2.6 ± 0.5 ^a(a)^	0.050 ± 0.006 ^b(a)^
AOST	3.9 ± 0.2	0.47 ± 0.01 ^b(b)^	−77 ± 5	70 ± 7	−3 ± 1	-	132 ± 5	incr.	108.4 ± 13.4 ^b(b)^	4.6 ± 0.4 ^a(b)^	0.043 ± 0.002 ^ab(a)^
COH	6.8 ± 0.1	0.60 ± 0.01 ^a^	−87 ± 4**b**	-	-	66 ± 0	99 ± 1**b**	incr.	278.4 ± 52.5 ^a^	11.0 ± 2.8 ^a^	0.039 ± 0.003 ^a^
AOH	7.7 ± 0.1	0.70 ± 0.01 ^b(a)^	−105 ± 3**b**	-	−90 ± 3**b**	17 ± 0 54 ± 3	107 ± 5	incr.	300.3 ± 57.3 ^a(a)^	9.9 ± 2.0 ^a(a)^	0.033 ± 0.003 ^a(a)^
AOHT	5.2 ± 0.2	0.78 ± 0.01 ^b(a)^	−91 ± 5**b**	58 ± 6	−55 ± 4	100 ± 1	131 ± 9	incr.	213.6 ± 21.2 ^a(a)^	8.0 ± 0.9 ^a(a)^	0.038 ± 0.003 ^a(a)^

**b**—broad peak, in some runs divided into two more or less separated peaks; incr.—the curve increases at higher temperatures, indicating a peak at temperatures higher than 150 °C; different superscripts (^a,b^) denote statistically significant (*p* < 0.05) differences among mean values within particular wood type groups (elm, oak sapwood, oak heartwood) according to Tukey’s HSD test: the first value relates to comparison between contemporary, archaeological untreated and treated wood, the second value (in brackets) relates to comparison between untreated and treated archaeological wood.

**Table 4:** The activation energy for γ-peaks and MTMS peaks of contemporary and archaeological untreated and treated wood samples.

Wood ID	MC (%)	Tan δ at Each Frequency			Activation Energy (kJ·mol^−1^)	
		1 Hz	5 Hz	10 Hz	γ Peak	MTMS
CE	8.3 ± 0.3	−100.7	−89.5	−86.2	39.2	-
AE	8.0 ± 0.4	−74.6	−66.6	−62.3	62.4	-
AET	4.2 ± 0.2	−86.2	−78.1	−73.8	55.1	152.2
COS	7.8 ± 0.2	−99.3	−85.8	−79.6	31.0	-
AOS	8.4 ± 0.4	−58.9	−54.1	−50.0	98.7	-
AOST	3.9 ± 0.2	−80.1	−67.7	−65.1	46.1	980.5
COH	6.7 ± 0.2	−84.9	−76.9	−66.7	38.4	-
AOH	7.7 ± 0.1	−98.3	−87.2	−75.8	28.1	-
AOHT	5.2 ± 0.2	−77.6	−63.7	−67.2	45.5	221.8

## Data Availability

The data underlying this article will be shared upon reasonable request from the corresponding author.

## References

[1] Florian M.-L.E. (1989). Scope and History of Archaeological Wood.

[2] Blanchette R.A. (2010). Microbial Degradation of Wood from Aquatic and Terrestrial Environments. Cult. Herit. Microbiol. Fundam. Stud. Conserv. Sci..

[3] Björdal C.G. (2012). Microbial Degradation of Waterlogged Archaeological Wood. J. Cult. Herit..

[4] Hoffmann P. (1986). On the Stabilization of Waterlogged Oakwood with PEG. II. Designing a Two-Step Treatment for Multi-Quality Timbers. Stud. Conserv..

[5] Unger A., Schniewind A., Unger W. (2001). Conservation of Wood Artifacts: A Handbook.

[6] Jensen P., Schnell U. The Implications of Using Low Molecular Weight PEG for Impregnation of Waterlogged Archaeological Wood Prior to Freeze Drying. Proceedings of the 9th ICOM Group on Wet Organic Archaeological Materials Conference.

[7] Bjurhager I., Ljungdahl J., Wallström L., Gamstedt E.K., Berglund L.A. (2010). Towards Improved Understanding of PEG-Impregnated Waterlogged Archaeological Wood: A Model Study on Recent Oak. Holzforschung.

[8] Almkvist G., Hocker E., Sahlstedt M., Museums S.M. (2013). Iron Removal from Waterlogged Wood.

[9] Almkvist G., Persson I. (2008). Degradation of Polyethylene Glycol and Hemicellulose in the Vasa. Holzforschung.

[10] Hocker E., Almkvist G., Sahlstedt M. (2012). The Vasa Experience with Polyethylene Glycol: A Conservator’s Perspective. J. Cult. Herit..

[11] Wagner L., Almkvist G., Bader T.K., Bjurhager I., Rautkari L., Gamstedt E.K. (2016). The Influence of Chemical Degradation and Polyethylene Glycol on Moisture-Dependent Cell Wall Properties of Archeological Wooden Objects: A Case Study of the Vasa Shipwreck. Wood Sci. Technol..

[12] Kaye B. (1995). Conservation of Waterlogged Archaeological Wood. Chem. Soc. Rev..

[13] Grattan D.W. (1982). A Practical Comparative Study of Several Treatments for Waterlogged Wood. Stud. Conserv..

[14] Hoffmann P. Sucrose for Waterlogged Wood: Not so Simple at All. Proceedings of the ICOM Committee for Conservation, 11th Triennial Meeting.

[15] Endo R., Kamei K., Iida I., Kawahara Y. (2008). Dimensional Stability of Waterlogged Wood Treated with Hydrolyzed Feather Keratin. J. Archaeol. Sci..

[16] Kennedy A., Pennington E.R. (2014). Conservation of Chemically Degraded Waterlogged Wood with Sugars. Stud. Conserv..

[17] McQueen C.M., Tamburini D., Lucejko J.J., Braovac S., Gambineri F., Modugno F., Colombini M.P., Kutzke H. (2017). New Insights into the Degradation Processes and Influence of the Conservation Treatment in Alum-Treated Wood from the Oseberg Collection. Microchem. J..

[18] Giachi G., Capretti C., Donato I.D., Macchioni N., Pizzo B. (2011). New Trials in the Consolidation of Waterlogged Archaeological Wood with Different Acetone-Carried Products. J. Archaeol. Sci..

[19] Broda M., Hill C.A.S. (2021). Conservation of Waterlogged Wood—Past, Present and Future Perspectives. Forests.

[20] Antonelli F., Galotta G., Sidoti G., Zikeli F., Nisi R., Petriaggi B.D., Romagnoli M. (2020). Cellulose and Lignin Nano-Scale Consolidants for Waterlogged Archaeological Wood. Front. Chem..

[21] Andriulo F., Giorgi R., Steindal C.C., Kutzke H., Braovac S., Baglioni P. (2017). Hybrid Nanocomposites Made of Diol-Modified Silanes and Nanostructured Calcium Hydroxide. Applications to Alum-Treated Wood. Pure Appl. Chem..

[22] Cavallaro G., Lazzara G., Milioto S., Parisi F., Ruisi F. (2017). Nanocomposites Based on Esterified Colophony and Halloysite Clay Nanotubes as Consolidants for Waterlogged Archaeological Woods. Cellulose.

[23] Cavallaro G., Milioto S., Parisi F., Lazzara G. (2018). Halloysite Nanotubes Loaded with Calcium Hydroxide: Alkaline Fillers for the Deacidification of Waterlogged Archeological Woods. ACS Appl. Mater. Interfaces.

[24] Cipriani G., Salvini A., Baglioni P., Bucciarelli E. (2010). Cellulose as a Renewable Resource for the Synthesis of Wood Consolidants. J. Appl. Polym. Sci..

[25] McHale E., Steindal C.C., Kutzke H., Benneche T., Harding S.E. (2017). In Situ Polymerisation of Isoeugenol as a Green Consolidation Method for Waterlogged Archaeological Wood. Sci. Rep..

[26] Salanti A., Zoia L., Zanini S., Orlandi M. (2016). Synthesis and Characterization of Lignin–Silicone Hybrid Polymers as Possible Consolidants for Decayed Wood. Wood Sci. Technol..

[27] Walsh Z., Janeček E.-R., Jones M., Scherman O.A. (2017). Natural Polymers as Alternative Consolidants for the Preservation of Waterlogged Archaeological Wood. Stud. Conserv..

[28] Walsh Z., Janeček E.-R., Hodgkinson J.T., Sedlmair J., Koutsioubas A., Spring D.R., Welch M., Hirschmugl C.J., Toprakcioglu C., Nitschke J.R. (2014). Multifunctional Supramolecular Polymer Networks as Next-Generation Consolidants for Archaeological Wood Conservation. Proc. Natl. Acad. Sci. USA.

[29] Walsh-Korb Z., Avérous L. (2019). Recent Developments in the Conservation of Materials Properties of Historical Wood. Prog. Mater. Sci..

[30] Zhou Y., Wang K., Hu D. (2019). High Retreatability and Dimensional Stability of Polymer Grafted Waterlogged Archaeological Wood Achieved by ARGET ATRP. Sci. Rep..

[31] Cavallaro G., Milioto S., Lazzara G. (2020). Halloysite Nanotubes: Interfacial Properties and Applications in Cultural Heritage. Langmuir.

[32] Lisuzzo L., Hueckel T., Cavallaro G., Sacanna S., Lazzara G. (2020). Pickering Emulsions Based on Wax and Halloysite Nanotubes: An Ecofriendly Protocol for the Treatment of Archeological Woods. ACS Appl. Mater. Interfaces.

[33] Parisi F., Bernardini F., Cavallaro G., Mancini L., Milioto S., Prokop D., Lazzara G. (2020). Halloysite Nanotubes/Pluronic Nanocomposites for Waterlogged Archeological Wood: Thermal Stability and X-ray Microtomography. J. Therm. Anal. Calorim..

[34] DeWolf H. (2007). Conservation Research Laboratory Instructions: Polymer Passivation (Silicone Oil) Conservation Treatment Based on Report by Helen Dewolf, 26 May 2004 and Modified by Donny L. Hamilton. Personal communication.

[35] Klosowski J.M., Smith C.W. (2000). Method of Conserving Waterlogged Materials.

[36] Smith C.W., Hamilton D.L. Treatment of Waterlogged Wood Using Hydrolyzable, Multi-Functional Alkoxysilane Polymers. Proceedings of the 8th ICOM Group on Wet Organic Archaeological Materials Conference.

[37] Broda M., Dąbek I., Dutkiewicz A., Dutkiewicz M., Popescu C.-M., Mazela B., Maciejewski H. (2020). Organosilicons of Different Molecular Size and Chemical Structure as Consolidants for Waterlogged Archaeological Wood—A New Reversible and Retreatable Method. Sci. Rep..

[38] Broda M. (2018). Biological Effectiveness of Archaeological Oak Wood Treated with Methyltrimethoxysilane and PEG against Brown-Rot Fungi and Moulds. Int. Biodeterior. Biodegrad..

[39] Broda M., Curling S.F., Spear M.J., Hill C.A. (2019). Effect of Methyltrimethoxysilane Impregnation on the Cell Wall Porosity and Water Vapour Sorption of Archaeological Waterlogged Oak. Wood Sci. Technol..

[40] Eder M., Arnould O., Dunlop J.W., Hornatowska J., Salmén L. (2013). Experimental Micromechanical Characterisation of Wood Cell Walls. Wood Sci. Technol..

[41] Green D.W., Winandy J.E., Kretschmann D.E. (1999). Mechanical Properties of Wood, Forest Products Laboratory. Wood Handbook—Wood as an Engineering Material.

[42] Hamdan S., Dwianto W., Morooka T., Norimoto M. (2000). Softening Characteristics of Wet Wood under Quasi Static Loading. Holzforschung.

[43] Hofstetter K., Gamstedt E.K. (2009). Hierarchical Modelling of Microstructural Effects on Mechanical Properties of Wood. A Review COST Action E35 2004–2008: Wood Machining–Micromechanics and Fracture. Holzforschung.

[44] Lenth C.A., Kamke F.A. (2007). Moisture Dependent Softening Behavior of Wood. Wood Fiber Sci..

[45] Lichtenegger H., Reiterer A., Stanzl-Tschegg S.E., Fratzl P. (1999). Variation of Cellulose Microfibril Angles in Softwoods and Hardwoods—A Possible Strategy of Mechanical Optimization. J. Struct. Biol..

[46] Mark R.E. (1967). Cell Wall Mechanics of Tracheids. Cell Wall Mechanics of Tracheids.

[47] Sheng-Zuo F., Wen-Zhong Y., Xiang-Xiang F.U. (2004). Variation of Microfibril Angle and Its Correlation to Wood Properties in Poplars. J. For. Res..

[48] Nguyen T.T., Xiao Z., Che W., Trinh H.M., Xie Y. (2019). Effects of Modification with a Combination of Styrene-Acrylic Copolymer Dispersion and Sodium Silicate on the Mechanical Properties of Wood. J. Wood Sci..

[49] Vorobyev A. (2017). Static and Time-Dependent Mechanical Behaviour of Preserved Archaeological Wood: Case Studies of the Seventeenth Century Warship Vasa. Ph.D. Thesis.

[50] Xie Y., Fu Q., Wang Q., Xiao Z., Militz H. (2013). Effects of Chemical Modification on the Mechanical Properties of Wood. Eur. J. Wood Wood Prod..

[51] Zhao S., Zhang Z., Sèbe G., Wu R., Virtudazo R.V.R., Tingaut P., Koebel M.M. (2015). Multiscale Assembly of Superinsulating Silica Aerogels Within Silylated Nanocellulosic Scaffolds: Improved Mechanical Properties Promoted by Nanoscale Chemical Compatibilization. Adv. Funct. Mater..

[52] Sun X., Jia X., Li F., Li J., Li J., Zhang C., Chen S., Cui J., Sun K., Zhang S. (2019). Effect of Poly-Methyltriethoxysilane on the Waterproof Property of Starch/Fiber Composites with Open Cell Structures. RSC Adv..

[53] Sun Z., Mingming W. (2019). Effects of Sol-Gel Modification on the Interfacial and Mechanical Properties of Sisal Fiber Reinforced Polypropylene Composites. Ind. Crops. Prod..

[54] Calabia B.P., Ninomiya F., Yagi H., Oishi A., Taguchi K., Kunioka M., Funabashi M. (2013). Biodegradable Poly(Butylene Succinate) Composites Reinforced by Cotton Fiber with Silane Coupling Agent. Polymers.

[55] Xie Y., Hill C.A., Xiao Z., Militz H., Mai C. (2010). Silane Coupling Agents Used for Natural Fiber/Polymer Composites: A Review. Compos. Part A Appl. Sci. Manuf..

[56] Olsson A.-M., Salmén L. (1997). The Effect of Lignin Composition on the Viscoelastic Properties of Wood. Nord. Pulp. Pap. Res. J..

[57] Placet V., Passard J., Perre P. (2007). Viscoelastic Properties of Green Wood across the Grain Measured by Harmonic Tests in the Range 0–95 C: Hardwood vs. Softwood and Normal Wood vs. Reaction Wood. Holzforschung.

[58] Sun N., Das S., Frazier C.E. (2007). Dynamic Mechanical Analysis of Dry Wood: Linear Viscoelastic Response Region and Effects of Minor Moisture Changes. Holzforschung.

[59] Kelley S.S., Rials T.G., Glasser W.G. (1987). Relaxation Behaviour of the Amorphous Components of Wood. J. Mater. Sci..

[60] Obataya E., Norimoto M., Tomita B. (2001). Mechanical Relaxation Processes of Wood in the Low-Temperature Range. J. Appl. Polym. Sci..

[61] Bjørkmann A., Salmén L. (2000). Studies on Solid Wood. II. The Influence of Chemical Modifications on Viscoelastic Properties. Cell. Chem. Technol..

[62] Salmén L., Stevanic J.S., Olsson A.-M. (2016). Contribution of Lignin to the Strength Properties in Wood Fibres Studied by Dynamic FTIR Spectroscopy and Dynamic Mechanical Analysis (DMA). Holzforschung.

[63] Stevanic J.S., Salmén L. (2020). Molecular Origin of Mechano-Sorptive Creep in Cellulosic Fibres. Carbohydr. Polym..

[64] Startsev O.V., Makhonkov A., Erofeev V., Gudojnikov S. (2017). Impact of Moisture Content on Dynamic Mechanical Properties and Transition Temperatures of Wood. Wood Mater. Sci. Eng..

[65] Gerhards C.C. (2007). Effect of Moisture Content and Temperature on the Mechanical Properties of Wood: An Analysis of Immediate Effects. Wood Fiber Sci..

[66] McCarthy C.J., Birkinshaw C., Pembroke J.T., Hale M. (1991). Dynamic Mechanical Analysis as a Technique for the Study of Fungal Degradation of Wood. Biotechnol. Tech..

[67] Curling S.F., Clausen C.A., Winandy J.E. (2002). Relationships between Mechanical Properties, Weight Loss, and Chemical Composition of Wood during Incipient Brown-Rot Decay. For. Prod. J..

[68] Ormondroyd G.A., Alfredsen G., Prabhakaran R.D., Curling S.F., Stefanowski B.K., Spear M.J., Gobakken L.R. (2017). Assessment of the Use of Dynamic Mechanical Analysis to Investigate Initial Onset of Brown Rot Decay of Scots Pine (*Pinus Sylvestris* L.). Int. Biodeter. Biodegr..

[69] Bledzki A.K., Faruk O., Huque M. (2002). Physico-Mechanical Studies of Wood Fiber Reinforced Composites. Polym. Plast. Technol. Eng..

[70] Sewda K., Maiti S.N. (2013). Dynamic Mechanical Properties of High Density Polyethylene and Teak Wood Flour Composites. Polym. Bull..

[71] Zhou Y., Fan M., Lin L. (2017). Investigation of Bulk and In Situ Mechanical Properties of Coupling Agents Treated Wood Plastic Composites. Polym. Test..

[72] Hristov V., Vasileva S. (2003). Dynamic Mechanical and Thermal Properties of Modified Poly (Propylene) Wood Fiber Composites. Macromol. Mater. Eng..

[73] Nkeuwa W.N., Riedl B., Landry V. (2014). UV-Cured Clay/Based Nanocomposite Topcoats for Wood Furniture. Part II: Dynamic Viscoelastic Behavior and Effect of Relative Humidity on the Mechanical Properties. Prog. Org. Coat..

[74] Fang L., Chang L., Guo W., Chen Y., Wang Z. (2014). Influence of Silane Surface Modification of Veneer on Interfacial Adhesion of Wood–Plastic Plywood. Appl. Surf. Sci..

[75] Broda M., Mazela B. (2017). Application of Methyltrimethoxysilane to Increase Dimensional Stability of Waterlogged Wood. J. Cult. Herit..

[76] Broda M., Frankowski M. (2017). Determination of the Content of Selected Elements in Medieval Waterlogged Oak Wood from the Lednica Lake—A Case Study. Environ. Sci. Pollut. R.

[77] Spear M.J., Broda M. (2020). Comparison of Contemporary Elm (*Ulmus* spp.) and Degraded Archaeological Elm: The Use of Dynamic Mechanical Analysis under Ambient Moisture Conditions. Materials.

[78] Rowell R.M., Ellis W.D. (1978). Determination of Dimensional Stabilization of Wood Using the Water-Soak Method. Wood Fiber Sci..

[79] Hill C., Altgen M., Rautkari L. (2021). Thermal Modification of Wood—A Review: Chemical Changes and Hygroscopicity. J. Mater. Sci..

[80] Brunauer S., Emmett P.H., Teller E. (1938). Adsorption of Gases in Multimolecular Layers. J. Am. Chem. Soc..

[81] Barrett E.P., Joyner L.G., Halenda P.P. (1951). The Determination of Pore Volume and Area Distributions in Porous Substances. I. Computations from Nitrogen Isotherms. J. Am. Chem. Soc..

[82] Broda M., Curling S.F., Frankowski M. (2021). The Effect of the Drying Method on the Cell Wall Structure and Sorption Properties of Waterlogged Archaeological Wood. Wood Sci. Technol..

[83] Glass S.V., Boardman C.R., Zelinka S.L. (2017). Short Hold Times in Dynamic Vapor Sorption Measurements Mischaracterize the Equilibrium Moisture Content of Wood. Wood Sci. Technol..

[84] Broda M., Mazela B., Radka K. (2019). Methyltrimethoxysilane as a Stabilising Agent for Archaeological Waterlogged Wood Differing in the Degree of Degradation. J. Cult. Herit..

[85] Popescu C.-M., Broda M. (2021). Interactions between Different Organosilicons and Archaeological Waterlogged Wood Evaluated by Infrared Spectroscopy. Forests.

[86] Schuerch C. (1963). Plasticizing Wood with Liquid Ammonia. Ind. Eng. Chem..

[87] Timar M.C., Mihai M.D., Maher K., Irle M. (2000). Preparation of Wood with Thermoplastic Properties, Part 1. Classical Synthesis. Holzforschung.

[88] Li Z., Jiang J., Lyu J. (2020). Moisture-Dependent Orthotropic Viscoelastic Properties of Chinese Fir Wood during Quenching in the Temperature Range of 20 to −120 °C. Holzforschung.

[89] Back E.L., Salmén N.L. (1982). Glass Transitions of Wood Components Hold Implications for Molding and Pulping Processes [Wood and Paper Materials]. TAPPI J. Tech. Assoc. Pulp Pap. Ind..

[90] Heijboer J. (1977). Secondary Loss Peaks in Glassy Amorphous Polymers. Int. J. Polym. Mater..

[91] Montes H., Mazeau K., Cavaillé J.Y. (1997). Secondary Mechanical Relaxations in Amorphous Cellulose. Macromolecules.

[92] Kim K.Y., Kim N.H., Nishinari K. (1991). Dielectric and Viscoelastic Properties of Cellulose Derivatives. Carbohydr. Polym..

[93] Ebringerová A., Heinze T. (2000). Xylan and Xylan Derivatives–Biopolymers with Valuable Properties, 1. Naturally Occurring Xylans Structures, Isolation Procedures and Properties. Macromol. Rapid Commun..

[94] Obataya E. (1996). Mechanical and Dielectric Relaxations of Wood in a Low Temperature Range. I. Relaxations Due to Methylol Groups and Adsorbed Water. Mokuzai Gakkaishi.

[95] Backman A.C., Lindberg K.A.H. (2001). Differences in Wood Material Responses for Radial and Tangential Direction as Measured by Dynamic Mechanical Thermal Analysis. J. Mater. Sci..

[96] Havimo M. (2009). A Literature-Based Study on the Loss Tangent of Wood in Connection with Mechanical Pulping. Wood Sci. Technol..

[97] Ashaduzzaman M., Hale M.D., Ormondroyd G.A., Spear M.J. (2020). Dynamic Mechanical Analysis of Scots Pine and Three Tropical Hardwoods. Int. Wood Prod. J..

[98] Jiang J., Lu J., Yan H. (2008). Dynamic Viscoelastic Properties of Wood Treated by Three Drying Methods Measured at High-Temperature Range. Wood Fiber Sci..

[99] Einfeldt J., Meißner D., Kwasniewski A. (2001). Polymerdynamics of Cellulose and Other Polysaccharides in Solid State-Secondary Dielectric Relaxation Processes. Prog. Polym. Sci..

[100] Sugiyama M., Obataya E., Norimoto M. (1998). Viscoelastic Properties of the Matrix Substance of Chemically Treated Wood. J. Mater. Sci..

[101] Goring D.A. (1963). Thermal Softening of Lignin, Hemicelluolose and Cellulose. Pulp Pap..

[102] Lin S.Y., Dence C.W. (2012). Methods in Lignin Chemistry.

[103] Olsson A.M., Salmén L. (1992). Viscoelasticity of in Situ Lignin as Affected by Structure: Softwood vs. Hardwood. ACS Symp. Ser. USA.

[104] Laborie M.-P., Salmén L., Frazier C.E. (2004). Cooperativity Analysis of the in Situ Lignin Glass Transition. Holzforschung.

[105] Fabiyi J.S., Ogunleye B.M. (2015). Mid-Infrared Spectroscopy and Dynamic Mechanical Analysis of Heat-Treated Obeche (Triplochiton Scleroxylon) Wood. Maderas Cienc. Technol..

[106] Jebrane M., Harper D., Labbé N., Sèbe G. (2011). Comparative Determination of the Grafting Distribution and Viscoelastic Properties of Wood Blocks Acetylated by Vinyl Acetate or Acetic Anhydride. Carbohydr. Polym..

[107] Pizzo B., Pecoraro E., Lazzeri S. (2018). Dynamic Mechanical Analysis (DMA) of Waterlogged Archaeological Wood at Room Temperature. Holzforschung.

[108] Boyd R.H. (1985). Relaxation Processes in Crystalline Polymers: Experimental Behaviour—A Review. Polymer.

